# A mini-review of the role of vesicular glutamate transporters in Parkinson’s disease

**DOI:** 10.3389/fnmol.2023.1118078

**Published:** 2023-05-11

**Authors:** Cheng Zhao, Chunyu Wang, Hainan Zhang, Weiqian Yan

**Affiliations:** ^1^Department of Neurology, The Second Xiangya Hospital, Central South University, Changsha, China; ^2^National Clinical Research Center for Geriatric Disorders, Xiangya Hospital, Central South University, Changsha, China; ^3^Department of Urology, Xiangya Hospital, Central South University, Changsha, China

**Keywords:** Parkinson’s disease, glutamate, vesicular glutamate transporters, excitatory amino acid transporters, glutamate receptors

## Abstract

Parkinson’s disease (PD) is a common neurodegenerative disease implicated in multiple interacting neurotransmitter pathways. Glutamate is the central excitatory neurotransmitter in the brain and plays critical influence in the control of neuronal activity. Impaired Glutamate homeostasis has been shown to be closely associated with PD. Glutamate is synthesized in the cytoplasm and stored in synaptic vesicles by vesicular glutamate transporters (VGLUTs). Following its exocytotic release, Glutamate activates Glutamate receptors (GluRs) and mediates excitatory neurotransmission. While Glutamate is quickly removed by excitatory amino acid transporters (EAATs) to maintain its relatively low extracellular concentration and prevent excitotoxicity. The involvement of GluRs and EAATs in the pathophysiology of PD has been widely studied, but little is known about the role of VGLUTs in the PD. In this review, we highlight the role of VGLUTs in neurotransmitter and synaptic communication, as well as the massive alterations in Glutamate transmission and VGLUTs levels in PD. Among them, adaptive changes in the expression level and function of VGLUTs may exert a crucial role in excitatory damage in PD, and VGLUTs are considered as novel potential therapeutic targets for PD.

## Introduction

Parkinson’s disease (PD) is a progressive neurodegenerative disease, implicated in multiple neurotransmitter pathways and autonomic nervous system that is associated with a range of clinical features (Schapira et al., 2017). Two types of clinical features are relied upon in its diagnosis: motor symptoms, including bradykinesia, stiffness, resting tremor, and postural and balance difficulties and non-motor symptoms, including autonomic dysfunction, sleep disturbances, behavioral changes, sensory abnormalities, and other unclassifiable symptoms (Kalia and Lang, 2015). The motor features are predominantly attributed to the formation of intracytoplasmic inclusion called Lewy bodies and the loss of dopamine (DA) neurons in the substantia nigra pars compacta (SNpc). The broad spectrum of non-motor symptoms of PD usually precede motor dysfunction. With the increasing awareness of the importance and presence of non-motor symptoms, PD is considered as a multisystem disorder involving various neurotransmitters in the brain (Klingelhoefer and Reichmann, 2017). Considering that most symptoms precede the complete loss of DA neurons in SNpc, it is likely that neuronal dysfunction precedes degeneration and other pathophysiological mechanisms drive the vulnerability of DA neurons.

The current standard drug therapy for PD is dopamimetic drugs, such as DA precursor levodopa (L-3,4-dihydroxyphenylalanine, L-DOPA), DA receptor agonists, and monoamine oxidase-B (MAO-B) inhibitors (Armstrong and Okun, 2020). In fact, the current DA replacement therapies neither improve most non-motor symptoms nor slow disease progression, highlighting the importance of studying the intervention of non-DA systems (Schapira et al., 2017). Indeed, various neurotransmitter systems are closely associated with the pathophysiology of PD (Sanjari et al., 2017). Among them, glutamate (Glu) is the most abundant transmitter in the central nervous system (CNS), and exerts vital effects on mediating the continuous feedback of basal ganglia circuits leading to DA dysregulation in the striatum (Wang et al., 2020). Emerging evidence suggests that glutamatergic transmission participates in the processes of PD, which is necessary for further study (Iovino et al., 2020; Pisanò et al., 2020; Lyu et al., 2021).

## Glutamate-glutamine cycle

**Figure 1:**
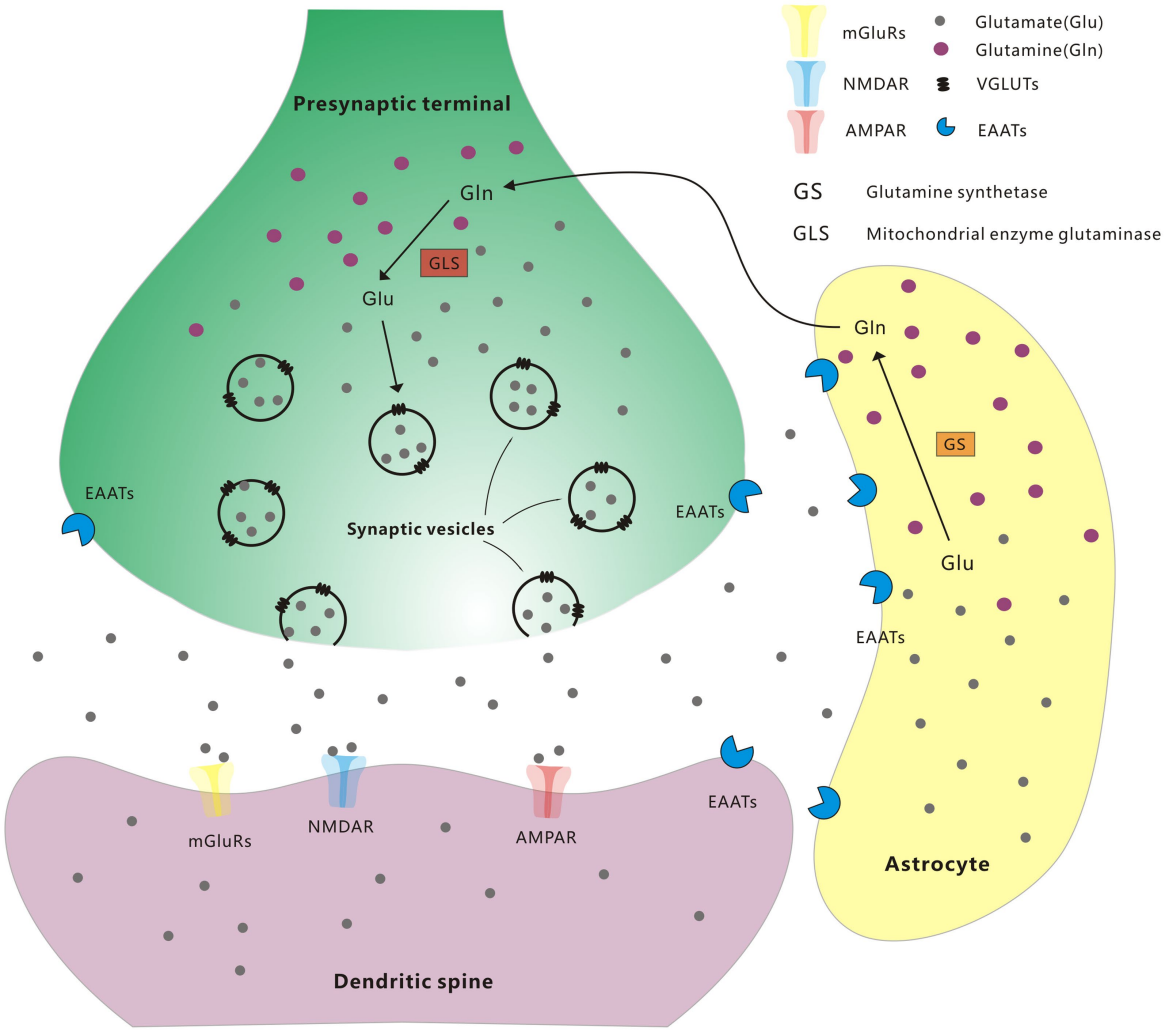
Glutamate-glutamine cycle.

Glutamate is the primary excitatory neurotransmitter in the brain and plays critical role in the control of neuronal activity. Glutamate is released from presynaptic terminals, and then it interacts with glutamate receptors (GluRs) on the plasma membrane of postsynaptic neurons. When triggered by glutamate, several types of GluRs work together to regulate excitatory postsynaptic neurotransmission (Plaitakis and Shashidharan, 2000). The specific receptors activated by glutamate can be divided into two main families: ionotropic and metabotropic GluRs (iGluRs and mGluRs). The iGluRs, including kainate receptors, α-amino-3-hydroxy-5-methyl-4-isoxazolepropionic acid (AMPA) receptors, and N-methyl-d-aspartate (NMDA) receptors, are multimeric ion channels in charge of rapid excitatory transmission in the mammalian CNS (Bigge, 1999). mGluRs are members of the G-protein-coupled receptor superfamily that contain eight receptor subtypes, inducing slow excitatory responses, which contribute to long-lasting effects in synaptic strength called long-term potentiation (LTP) or long-term depression (LDP; Ferraguti and Shigemoto, 2006). In presynaptic neurons, glutamine is converted to glutamate by mitochondrial enzyme glutaminase, and then packaged by vesicular glutamate transporters (VGLUTs) into synaptic vesicles, followed by releasing into the synaptic cleft by stimulation (El et al., 2011). Glutamate in the synaptic cleft is removed by excitatory amino acid transporters (EAATs) situated on the neuronal plasma membrane, and is also able to transport glutamate to astrocyte or back to presynaptic terminals. Within the astrocyte, glutamate is transformed into glutamine by glutamine synthetase (GS), and then transported back to neurons sequentially through glutamine transporters on the membrane of astrocytes and neurons (Andersen and Schousboe, 2022; Figure 1). All these transporters facilitate the transport of glutamate, ensuring that glutamate is maintained with the appropriate concentration in the correct compartment.

Extracellular glutamate concentrations are mainly regulated via EAATs with high affinity. EAATs have five characterized mammalian subtypes, including glutamate/aspartate transporter (GLAST, also named EAAT1), glutamate transporter-1 (GLT-1, also named EAAT2), excitatory amino acid carrier-1 (EAAC1, also named EAAT3), EAAT4, and EAAT5. EAATs are proved to maintain the balance of extracellular glutamate concentrations and protect neurons from harmful overstimulation of GluRs (Magi et al., 2019). Noteworthy, Glutamate concentration is also regulated by modulating glutamate internalization into synaptic vesicles through VGLUTs (Shigeri et al., 2004). The expression and function of VGLUTs play an important role in glutamate release in presynaptic regions. The expression level of VGLUTs in each synaptic vesicle indicates the relative intensity of presynaptic glutamatergic innervation and control the quantal size of glutamatergic transmission (Daniels et al., 2006; Liguz-Lecznar and Skangiel-Kramska, 2007). The involvement of GluRs and EAATs in the pathophysiology of PD has been widely studied, but little is known about the role of VGLUTs in PD. Therefore, given that a comprehensive understanding of the pathophysiology and therapeutic targets of VGLUTs for PD can help in the development of new therapeutic approaches for PD. This article highlights the role of VGLUTs in neurotransmitter and synaptic communication, as well as the massive alterations in Glutamate transmission and VGLUTs levels in PD.

## Distribution and molecular pharmacology of VGLUTs

Three subtypes of VGLUTs have been identified and characterized, named VGLUT1-3, which are encoded by solute vector gene *Slc17a6-8* (Bellocchio et al., 2000; Takamori et al., 2000). The distributions of the three VGLUTs barely overlap, delineating 3 complementary glutamate systems that VGLUT1 (*Slc17a7*) and VGLUT2 (*Slc17a6*) exert dominating neurophysiological impacts on almost all central neuronal circuits, whereas VGLUT3 (*Slc17a8*) participates in local transmission regulation (Fremeau et al., 2001; Schäfer et al., 2002). VGLUT1 and VGLUT2 are specific markers of glutamatergic neurons, which are co-expressed in most of the brain region, including the cerebral cortex, occipital lobe, frontal lobe, temporal lobe, cerebellum, amygdala, medulla, hippocampus, and putamen. Additionally, VGLUT2 is also expressed in many other parts of the brain, including substantia nigra, caudate nucleus, thalamus, subthalamic nucleus, and spinal cord (Fremeau et al., 2001; Kaneko and Fujiyama, 2002; Herzog et al., 2004; Hackett et al., 2011). Notably, VGLUT2 is the only vesicular glutamate transporter expressed in transgenic ventral tegmental area (VTA)/substantia nigra dopamine neurons (Kouwenhoven et al., 2020). VGLUT3 is only expressed in a few glutamate neurons in raphe nuclei, cerebral cortex, and cochlear inner hair cells (IHCs; Gras et al., 2002; Ruel et al., 2008; Hioki et al., 2010). VGLUT3 predominantly exists in scattered group of “non-glutamatergic” neurons, including cholinergic interneurons (ChIs) in the ventral and dorsal striatum, GABAergic neurons in the olfactory bulb, GABAergic cortical and hippocampal interneurons, and 5-hydroxytryptaminergic olecranon neurons (Somogyi et al., 2004; Gras et al., 2008; Tatti et al., 2014; Fasano et al., 2017). Moreover, VGLUT3 is widely expressed outside the brain, particularly in cochleae, retina, and spinal cord (Lee et al., 2014; Cheng et al., 2017).

These three transporters (VGLUT1-3) show strong sequence homology, particularly in the transmembrane structural domains that constitute the translocation pathway (Bellocchio et al., 2000; Takamori et al., 2000). In addition, Inherent transport activities are found no difference among them. VGLUTs have relatively low affinity [K(m) = 1–2 mM] for glutamate, which make it difficult to identify VGLUTs inhibitors with high affinity (Thompson et al., 2005). VGLUTs are highly selective for glutamate which make them selectively targeted, thus the effect of pharmacological manipulations by small molecules do not disrupt other transport phenomena, like EAATs or GluRs (Thompson and Chao, 2020). In contrast to most plasma membrane transporters, the VGLUTs, like other endosomal neurotransmitter transporters, rely on the proton electrochemical gradient across the synaptic vesicle membrane generated by vacuolar-type H + -ATPase (V-ATPase; Blakely and Edwards, 2012). The difference is vesicular neurotransmitter transporters are driven by proton exchange and thus depend on the chemical component of ∆μH+ (∆pH), glutamate uptake by VGLUTs depends on the membrane potential (∆ψ), suggesting a mechanism of facilitated diffusion (Maycox et al., 1988). Subsequently, chloride (Cl^−^) ions are reported to greatly stimulate glutamate uptake by synaptic vesicles *in vitro* (Wolosker et al., 1996). Increasing studies soon confirmed these initial results (Martineau et al., 2017; Eriksen et al., 2020; Chen et al., 2021). In conclusion, the vacuolar-type H + -ATPase generate transmembrane proton electrical gradient provide powers for VGLUTs, while VGLUTs binding with chloride, potassium, and protons regulate VGLUTs activity as well (Pietrancosta et al., 2020).

## Molecular mechanism of VGLUTs in PD

In recent years, studies have indicated the subtle, but important, participation of VGLUTs-dependent glutamate/DA co-transmission and its roles in the regulation of different brain functions and dysfunctions (Buck et al., 2021c, 2022). In-depth study in the molecular mechanism of VGLUTs could result in decisive breakthroughs in the treatment of PD.

## VGLUT1 and Parkinson’s disease

Plenty of cortical excitatory neurons express VGLUT1, which represents a major isoform in the brain, accounting for about 80% of total glutamatergic vesicular transports (Fremeau et al., 2001). Glutamatergic neurotransmission in the striatum has been involved in the progression of PD. Biphasic and bilateral alterations in the levels of VGLUT1 and VGLUT2 protein expression of the striatum in hemiparkinsonian rats suggest significant time-dependent changes in glutamatergic neurotransmission from both types of striatal afferents (Massie et al., 2010). Study has revealed that glutamate is significantly reduced in synaptic vesicle-enriched membrane fractions of VGLUT1−/− mice, the absence of VGLUT1 may alter the ability of releasing glutamate from nerve endings (Wojcik et al., 2004). The glutamatergic pathways exert significant functions in neuronal circuits related to PD.

The progressive degeneration of DA-capable cells in SNpc results in the imbalance within the cortico-basal ganglia loop, related to aberrant glutamatergic innervation in the brain (Orieux et al., 2000; Cilia et al., 2009). It has been reported that compared to controls, the protein level of VGLUT1 is decreased in the prefrontal cortex (PFC) of PD patients, revealing that VGLUT1 exerts the significant effects on glutamatergic damage in patients with PD (Kashani et al., 2007). Whereas, the study of Raju et al. has revealed the significantly increased total density of VGLUT1 in the striatum of PD monkeys after treatment with 1-methyl-4-phenyl-1,2,3,6-tetrahydropyridine (MPTP; Raju et al., 2008). In addition, El Arfani et al. have also noted alterations of different glutamate transporter expression levels in the bilaterally-lesioned 6-hydroxydopamine (6-OHDA) rat model, bilateral SNpc lesions inhibit the expression of VGLUT1 and showed a remarkable change after 2 weeks of injury in the striatum, but no significant changes were observed in the motor cortex (El et al., 2015). In a PD mouse model, the MPTP-induced expression of VGLUT1 protein is elevated in the medial PFC with loss of DA, while the expression of VGLUT1 in the dorsolateral striatum is significantly decreased (Pflibsen et al., 2015). These findings indicate that the remarkable variations in glutamate delivery transported by VGLUT1 may related to motor and cognitive deficits of PD.

Increasing evidence has demonstrated that as a treatment method for PD, electroacupuncture (EA) is able to facilitate the improvement of motor function in PD (Jia et al., 2010). In clinical practice, the subthalamic nucleus (STN) is considered as a pivotal target of deep brain stimulation for PD treatment, and VGLUT1 is closely involved in glutamate regulation of the cortical STN (Wang et al., 2018). Electroacupuncture can reverse 6-OHDA-induced VGLUT1 expression reduction in the STN (Zheng et al., 2019). Electroacupuncture promotes VGLUT1 expression in the ipsilateral STN and improves motor symptoms in PD rats, indicating that the overexpression of VGLUT1 in the STN may be associated with the role of EA in motor symptoms of PD via the cortical-STN pathway.

## VGLUT2 and Parkinson’s disease

VGLUT1 and VGLUT2 have complementary distributions throughout the adult brain. VGLUT2 predominantly exists in glutamate neurons of subcortical brain regions, such as SNpc, VTA, and thalamus (Kashani et al., 2007). Endogenous VGLUT2 has also been proved to express in a subpopulation of midbrain DA neurons (Kawano et al., 2006; Yamaguchi et al., 2011, 2015). A few (<20%) DA neurons in midbrain express detectable levels of VGLUT2 in the adulthood. However, the rate of co-localization of VGLUT2 and DA may increase during development (Mendez et al., 2008; Bérubé-Carrière et al., 2009). Consistently, it has been shown more than 90% of SNpc DA neurons presented a reporter indicative of past expression of VGLUT2 based on a fate mapping strategy (Steinkellner et al., 2018). VGLUT2 is expressed in SNpc DA neurons early in life, while most of these DA neurons present decreased expression of VGLUT2 at maturity. VGLUT2 facilitates the encapsulation of glutamate into synaptic vesicles *in vitro* (Kouwenhoven et al., 2020). Study have shown that VGLUT2 contributes to vesicular DA loading by increasing the pH gradient of vesicles (or vesicular hyper-acidification; Aguilar et al., 2017).

VGLUT2 functionally regulates the core co-release of glutamate and DA from VGLUT2+ DA neurons. VGLUT2 is the dominant subtype of VGLUTs existed in midbrain DA neurons (Bérubé-Carrière et al., 2009; Chuhma et al., 2009; Morales and Root, 2014; Morales and Margolis, 2017). Furthermore, VGLUT2 selectively deleted from DA neurons may influence the growth and survival of DA neurons in cell culture and development *in vivo* (Hnasko et al., 2010). Similarly, the loss of DA neurons in SNpc caused by overexpression of VGLUT2 is accompanied by changes in motor behavior of mice (Steinkellner et al., 2018). In general, these behavioral abnormalities are strongly linked to decreased striatal DA neurotransmission in involved hemispheres. Postmortem brain tissues from PD patients exhibit marked variations in expression of VGLUTs in the cerebral cortex and striatum, indicating the important role of VGLUTs in PD (Kashani et al., 2007). Previous research has shown the essential roles of VGLUT2 expression in DA neurons in normal emotional responses as well as behavioral activation mediated by psychostimulant (Birgner et al., 2010). Several studies have shown that downregulation of VGLUT2 expression exclusively in the STN of mice leads to reduced postsynaptic activity and behavioral hyperlocomotion, due to the strong modifications in both the STN and the striatum DA system (Schweizer et al., 2014, 2016). Moreover, MPTP-treated mouse model of PD has increased expression of VGLUT2 in the striatum in comparison to controls (Pflibsen et al., 2015).

VGLUT2 is deemed to promote the survival of DA neurons. Shen et al. have reported that VGLUT2 selectively deleted from DA neurons obviously increases the susceptibility of DA neurons to neurotoxin MPTP, and furthermore, upregulation of VGLUT2 in DA neurons prevented this vulnerability in VGLUT2 conditional Knock-out (KO) mice (Shen et al., 2018). This finding suggests that the absence or reduction of VGLUT2 expression in several DA neurons may be considered as a novel risk factor for the occurrence and progression of DA neurodegeneration in PD. Therefore, restoring the expression of VGLUT2 in DA neurons may be a potential and novel therapeutic method for PD or other neurodegenerative diseases. In contrast, Steinkellner et al. reported that the proportion of DA neurons expressing VGLUT2 approximately doubled after 6-OHDA injection in the striatum. Notably, although the neurotoxicity of 6-OHDA reduced the total DA neurons, the number of DA neurons containing elevated VGLUT2 transcripts was definitely increased, suggesting that 6-OHDA caused upregulation of VGLUT2 in transcriptional levels in adult SNpc DA neuron cells (Steinkellner et al., 2018). They further demonstrated that VGLUT2+ DA neurons enriched in surviving neurons in α-synuclein-induced dopaminergic neuronal injury, and VGLUT2 expression was found upregulated in brain tissue of PD patients (Steinkellner et al., 2022). This result is consistent with previous studies which revealed that neonatal striatal lesions or 6-OHDA treatment promote the expression of VGLUT2 (Dal Bo et al., 2008; Bérubé-Carrière et al., 2009). Buck et al. also found that the subpopulation of VGLUT2+ DA neurons are relatively protected from rotenone neurotoxicity (Buck et al., 2021a). In summary, the effects of different neurotoxicants produced an analogous change. The above findings implied that the neurotoxins-induced upregulation of the glutamatergic machinery in VTA and SNpc neurons and their projections may be part of a broader neuroprotective mechanism. Moreover, Buck et al. demonstrated that female drosophila has elevated expression level of dVGLUT in DA neurons compared with male drosophila, and this finding is highly conserved across species, including flies, rodents, and humans. Moreover, they found that reducing the expression of dVGLUT in DA neurons eliminates females’ better resilience to DA neuron loss throughout aging. dVGLUT is the core role in the selective DA neuron vulnerability to sex- and age-related DA neurodegeneration (Buck et al., 2021b).

Noteworthy, Heterologous expression of VGLUT2 with a sustained or high level is toxic to DA neurons, while endogenous expression of VGLUT2 with a low level might exert a protective effect (Buck et al., 2022). Glutamate co-entry through the vesicle of VGLUTs is able to drive VMAT2-mediated exchange, which can elevate the amount of DA and other cationic transmitters, as well as contribute to isolate toxic VMAT2 substrates, including 1-methyl-4-phenylpyridine (MPP^+^) or 6-OHDA, away from sensitive cellular compartments (Dal Bo et al., 2008; Descarries et al., 2008). Therefore, the expressing of VGLUT2 is likely to explain the enhancive resistance of DA neurons to neurotoxins. DA-depletion has no influence in the expression of VGLUT1 and VGLUT3, but VGLUT2 expression is conspicuously reduced in almost all basal ganglia structures (Favier et al., 2013). High-frequency stimulation of the subthalamic nucleus (STN-HFS) can reverse the decrease in VGLUT2 expression, which provides evidence for the involvement of VGLUT2 in the regulation of basal ganglia circuitry, suggesting that VGLUT2 exert an important role in alleviating motor symptoms in PD (Favier et al., 2013). Since VGLUT2 is the only VGLUT produced by STN glutamatergic projections to SNpr, we speculate that during STN-HFS, information transmission through the trans-thalamic pathway has not been fully interrupted, despite its roles in the expression of VGLUT2 in SNpr are likely mediated by regulation of thalamic afferents.

## VGLUT3 and Parkinson’s disease

Both VGLUT1 and VGLUT2 are originally known as “typical” cortical and subcortical VGLUTs. However, VGLUT3 predominantly exists in scattered group of “non-glutamatergic” neurons, or only expresses in a few glutamate neurons (El et al., 2011). VGLUT3 represents a unique modulator of glutamate release from both non-glutamatergic and glutamatergic neurons in the brain (Favier et al., 2021). Although the distribution and quantity of VGLUT3 is limited, it plays a vital role in regulation glutamate signaling and thus modulates the activity of neural microcircuits (Favier et al., 2021). Currently, few studies have explored the role of VGLUT3 in PD, most studies focus on revealing the critical role of VGLUT3 in levodopa-induced dyskinesia (LID), which usually occurs in PD patients with long-term L-DOPA treatment (Divito et al., 2015; Gangarossa et al., 2016).

Reduction of DA transmission triggers profound adaptive changes in DA-sensitive brain structures, particularly the dorsal striatum. Striatal cholinergic interneurons (ChIs) are the main source of acetylcholine in the striatum. The increased striatal cholinergic tone is the main pathogenic mechanism among all the alterations associated with PD, as ChIs potently regulates local striatal microcirculation, which attracts intensive researchers to establish anticholinergic treatment for PD (McKinley et al., 2019). However, regulation of striatal ChIs directly leads to alterations in local striatal glutamate transmission (Tubert et al., 2016). VGLUT3 is highly expressed in striatum, and plays an important role in ChIs-mediated glutamate release (Gras et al., 2008). Meanwhile, the activity of GABAergic medium spiny neurons (MSNs) and fast-spiking GABAergic interneurons (FSIs) are related to the VGLUT3-dependent glutamate transmission (Favier et al., 2021). VGLUT3-KO mice show circadian-dependent hyperlocomotor activity, while conditional deletion of VGLUT3 from ChIs does not alter evoked DA release in the striatum or baseline locomotor activity (Divito et al., 2015). Loss of DA in 6-OHDA-lesioned mice is accompanied by increased expression of VGLUT3 and vesicular acetylcholine transporter (VAChT) in the striatum, and the VAChT levels remain high whereas the VGLUT3 expression decreases in LID mice (Gangarossa et al., 2016).

## Conclusion

In this review, we summarized the distribution and functional characteristics of VGLUTs in the brain, and indicate the pivotal influence of glutamate transmission in the functional organization of neuronal circuits in PD, as well as the massive alterations in glutamate transmission and VGLUTs levels in PD. Among them, adaptive changes in the expression level and function of VGLUTs may exert a crucial role in excitatory damage in PD, and VGLUTs are considered as novel potential therapeutic targets for PD. Notably, recent years, there have been several authoritative studies on VGLUT2, and emerging evidence highlights that the balance of VGLUT2 expression in select DA neuronal populations may be a novel identified risk factor or therapeutic target in the progression of PD or other neurodegenerative diseases. Taken together, the structure, function, and regulatory mechanisms of VGLUTs will be a promising area of research in PD clinical practice.

## Author contributions

WY contributed to the conception of the study. CZ contributed to search and organize literatures. WY and CZ wrote the manuscript. CW and HZ contributed equally to this work in administrative and material support. All authors contributed to the article and approved the submitted version.

## Funding

This research was supported by the grant of National Natural Science Foundation of China (no. 81801123) to WY, Natural Science Foundation of Hunan Province (no. 2023JJ40865) to WY and Natural Science Foundation of Hunan Province (no. 2021JJ41055) to CZ.

## Conflict of interest

The authors declare that the research was conducted in the absence of any commercial or financial relationships that could be construed as a potential conflict of interest.

## Publisher’s note

All claims expressed in this article are solely those of the authors and do not necessarily represent those of their affiliated organizations, or those of the publisher, the editors and the reviewers. Any product that may be evaluated in this article, or claim that may be made by its manufacturer, is not guaranteed or endorsed by the publisher.
